# Elevated blood pressure and illness beliefs: a cross-sectional study of emergency department patients in Jamaica

**DOI:** 10.1186/s12245-018-0187-6

**Published:** 2018-05-30

**Authors:** Taneisha T. Wilson, Jean Williams-Johnson, Maxine Gossel-Williams, Elizabeth M. Goldberg, Rainford Wilks, Shuvra Dasgupta, Georgiana M. Gordon-Strachan, Eric W. Williams, Philip D. Levy

**Affiliations:** 1 Alpert School of Medicine, Brown University, Emergency Medicine, Rhode Island Hospital, 55 Claverick St. #2, Providence, RI 02903 USA; 2 University Emergency Medicine Foundation, Kingston, Jamaica; 3 University Hospital, University of the West Indies, Mona, Kingston, Jamaica; 4 The University Hospital of the West Indies, Mona, Kingston, West Indies Jamaica; 5 Department of Basic Medical Sciences, University of the West Indies, Mona, Kingston, Jamaica; 6 The University of the West Indies, Mona, Kingston, West Indies Jamaica; 7 Tropical Medicine Research Institute, University of the West Indies, Mona, Kingston, Jamaica; 8 Department of Emergency Medicine, Wayne State University, Detroit, MI USA; 9 Emergency Medicine, 6G4 University Health Center, Detroit, MI 48201 USA

**Keywords:** Hypertension, Illness beliefs, Afro-Caribbean, Medication self-efficacy, Emergency department

## Abstract

**Background:**

Elevated blood pressure (BP) is common among emergency department (ED) patients. While some data exist on the association between ED BP and hypertension (HTN) in the USA, little is known about this relationship in Afro-Caribbean nations. The aim of the study was to evaluate the relationship between elevated systolic BP in the ED and a previous diagnosis of HTN, accounting for potential factors that could contribute to poor HTN control among those with a previous diagnosis: socioeconomic status, health-seeking behavior, underlying HTN illness beliefs, medication adherence, and perceived adherence self-efficacy.

**Methods:**

This was a cross-sectional survey over 6 weeks, from November 19 through December 30, 2014. Those surveyed were non-critically ill or injured adult ED patients (≥ 18 years) presenting to an Afro-Caribbean hospital. Descriptive statistics were derived for study patients as a whole, by HTN history and by presenting BP subgroup (with systolic BP ≥ 140 mmHg considered elevated). Data between groups were compared using chi-square and *t* tests, where appropriate.

**Results:**

A total of 307 patients were included: 145 (47.2%) had a prior history of HTN, 126 (41.4%) had elevated BP, and 89 (61.4%) of those presenting with elevated blood pressure had a previous diagnosis of HTN. Those with less formal education were significantly more likely to present with elevated BP (52.1 vs. 28.8% for those with some high school and 19.2% for those with a college education; *p* = 0.001). Among those with a history of HTN, only 56 (30.9%) had a normal presenting BP. Those with a history of HTN and normal ED presenting BP were no different from patients with elevated BP when comparing the in duration of HTN, medication compliance, location of usual follow-up care, and HTN-specific illness beliefs.

**Conclusions:**

In this single-center study, two out of every five Jamaican ED patients had elevated presenting BP, the majority of whom had a previous diagnosis of HTN. Among those with a history of HTN, 60% had an elevated presenting BP. The ED can be an important location to identify patients with chronic disease in need of greater disease-specific education. Further studies should evaluate if brief interventions provided by ED medical staff improve HTN control in this patient population.

**Electronic supplementary material:**

The online version of this article (10.1186/s12245-018-0187-6) contains supplementary material, which is available to authorized users.

## Background

The Jamaican Health and Lifestyle survey of 2007–2008 estimated that approximately 25% of the Jamaican population aged 15–74 had high blood pressure (BP); however, only 50% were aware that they had elevated blood pressure [1–3]. For each 20 mmHg systolic or 10 mmHg diastolic increase in blood pressure (BP) above normal, there is a doubling in risk of cardiovascular, cerebrovascular, and renovascular disease [4–7].

In the USA in 2005, there were 3.3 million high BP-related emergency department (ED) visits [8]. Recent data suggest that the majority of patients with elevated BP in the ED carry a true diagnosis of hypertension (HTN) [9, 10]. In the USA and other locations worldwide, the ED often serves as the safety net for persons with chronic diseases such as HTN and may be an important location to help prevent adverse consequences associated with poor BP control [9, 11, 12].

Anecdotal evidence suggests that there are a significant number of patients who present to Jamaican EDs with elevated BP, those both with and without a prior outpatient diagnosis of HTN. The same may be true for Afro-Caribbean locations, but epidemiological data are lacking. The ED could play an important role in increasing knowledge surrounding how to achieve well-controlled HTN by providing brief education and referral [13]. The ED is often the only source of medical care for those who are underserved [14, 15]. For this reason, emergency medicine clinicians play an important role in improving population BP control [16].

This study was designed to evaluate patients who present to the ED with elevated BP in a representative Afro-Caribbean institution and to evaluate, among patients with known HTN, the relationship between elevated BP and potential contributory factors such as socioeconomic status, health-seeking behavior, and underlying HTN illness beliefs.

## Methods

### Setting and subjects

This cross-sectional survey was conducted over a 6-week period from November 19 through December 30, 2014, at an Afro-Caribbean hospital. The hospital is a large urban teaching hospital located in the Kingston Metropolitan Area. It is affiliated with the University, and the Emergency Medicine division treats approximately 53,000 patients each year.

Patients ≥ 18 years old who presented to the ED were considered eligible for participation in this study. Those who presented in cardiac arrest, were suffering from trauma requiring immediate operative intervention, or were too ill to provide consent were excluded. This study was approved by the Ethics Committee of the University and all subjects were required to provide informed consent prior to enrollment.

### Data collection

Research assistants administered the survey for approximately 9 h each day. The ED is divided into three sections: a fast track area, an intermediate care area, and an acute care area. Patients are triaged based on an assessment by a triage nurse and physician. All patients have an initial BP measured by the registered nurse at triage. During the study collection period, research assistants spent 3 h in each section of the ED. They rotated the starting section each day as follows: day 1: fast track (3 h), intermediate (3 h), acute care (3 h); day 2: intermediate (3 h), acute care (3 h), fast track (3 h); and day 3: acute care (3 h), fast track (3 h), intermediate (3 h).

Research assistants approached patients immediately after evaluation by the treating physician. Once consent was obtained, BP, medical history, and socioeconomic information were collected for all patients. For purposes of the study, we defined a triage nurse measured systolic BP among patients with known HTN, ≥ 140 mmHg as elevated BP. Patients with a known or documented history of HTN, whether they were on antihypertensive medication at the time of enrollment or not, were considered as having a history of HTN. For those with a history of HTN, disease-specific illness beliefs and medication adherence were collected, along with perspectives on medication adherence using a previously validated self-efficacy scale [17, 18].

### Statistical analysis

Descriptive statistics were derived for study patients as a whole, by presenting BP subgroups and by HTN history. Data between the groups were compared using chi-square analysis and *t* tests where appropriate using STATA 14 [19].

## Results

**Fig. 1:**
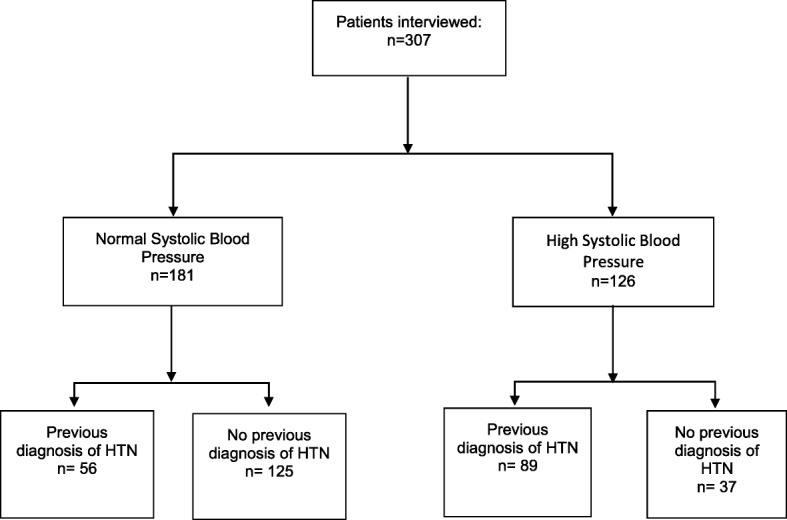
Study flow diagram. HTN = hypertension

A total of 307 patients were enrolled during the study period, 59.0% had normal BP. Forty-one percent had an elevated presenting BP while 61.4% had a previous diagnosis of HTN (Fig. 1; Table 1). Age was significantly different among those presenting with elevated BP (59.5 [± 17.6] years vs. 48.9 [± 21.6] years; *p* < 0.0001). Those with less formal education were significantly more likely to present with elevated BP (52.1 vs. 28.8% for those with some high school and 19.2% for those with a college education; *p* = 0.01). Self-reported annual income was not significantly different among those with elevated BP compared to those with normal BP ($17,746 [± 21,648] JD vs. $15,659 [± 14,538] JD; *p* = 0.26) (Table 2).

**Table 1:** Comparing normal vs. elevated triage systolic blood pressure and history of previously diagnosed hypertension

	Normal SBP, *n* (%) 181 (59.0)	Elevated SBP, *n* (%) 126 (41.0)	Total, *n* (%) 307 (100.0)
No history of HTN	125 (77.2)	37 (22.8)	167 (100.0)
History of HTN	56 (38.6)	89 (61.4)	145 (100.0)

**Table 2:** Demographic characteristics of Jamaican ED patients presenting with normal vs. elevated presenting systolic BP

	Normal SBP *N =* 181 (59.0%)	Elevated SBP *N =* 126 (41.0%)	*p* value
Age, years (± SD)	48.9 (21.6)	59.5 (17.6)	0.0001
History of HTN, *n* (%)	56 (38.6)	89 (61.4)	0.0001
SBP, m (± SD)	119.8 (13.9)	167.1 (22.0)	0.0001
Income, JD (± SD)	17,746 (21648)	15,659 (14538)	0.26
Gender, *n* (%)			0.26
Women	96 (58.2)	75 (64.4)	
Men	85 (41.9)	51 (35.6)	
Education, *n* (%)			0.01
Less than HS	64 (34.7)	65 (52.1)	
HS	76 (42.4)	36 (28.8)	
Some college	40 (22.9)	24 (19.2)	
Employment, *n* (%)			0.45
Unemployed	103 (57.1)	79 (62.9)	
Employed	62 (33.9)	34 (27.1)	
Retired	16 (0.09)	13 (0.1)	

*ED* emergency department, *SBP* systolic blood pressure, *SD* standard deviation, *%* percent, *JD* Jamaican dollars, *HS* high school

Among those with a history of HTN (*n* = 145), only 38.6% had a normal BP (Table 3). The mean length of HTN diagnosis was similar among those both with a normal BP (14.7 [± 11.2 years) and without (13.2 [± 12] years) *p* = 0.239). Among those with an elevated presenting BP, anti-hypertensives were prescribed to 62.7% of the participants compared to 37.3% of those without elevated BP on presentation. Self-reported medication adherence was high with no differences between groups (77.0% for those with normal BP vs. 82.2% in those with elevated BP; *p* = 0.37). The majority of patients with a previous diagnosis of HTN received their BP management at private physician offices with no difference by group. HTN-specific illness and medication beliefs (Table 4) were similar among patients with a history of HTN who had normal vs. elevated systolic BP. There were also no differences in perceived medication self-efficacy among known hypertensive patients with normal vs. elevated BP (Table 5).

**Table 3:** Hypertension care among those with a previous diagnosis of hypertension

	Normal SBP *N* = 56 (38.6)	Elevated SBP *n* (%) 89 (61.4)	*p* value
Time since HTN diagnosis, years (SD)	14.7 (11.2)	13.2 (12.0)	0.239
Prescribed home BP medications, *n* (%)^a^	55 (37.3)	90 (62.7)	–
BP medication adherence, *n* (%)			0.370
No	12 (21.4)	16 (17.5)	
Yes	42 (77.0)	74 (82.5)	
Location of HTN care, *n* (%)			0.370
Health center	9 (16.2)	10 (11.6)	
Private doctor	35 (64.5)	50 (56.0)	
Hospital clinic	5 (9.26)	17 (19.1)	
ED	10 (18.5)	13 (14.8)	

*SBP* systolic blood pressure, *HTN* hypertension, *SD* standard deviation, *ED* emergency department

^a^All patients reported home anti-hypertensive prescription

**Table 4:** Patient responses about HTN illness beliefs among those with a previous diagnosis of hypertension: comparing those presenting with and without elevated BP

	Normal SBP on presentation mean (95% CI)	Elevated SBP on presentation mean (95% CI)	*p* value
Is an illness that I cannot influence by my behavior	2.64 (2.49–2.78)	2.71 (2.51–2.90)	0.56
Is something I go “in” and “out” of	2.83 (2.71–2.95)	2.87 (2.67–3.01)	0.72
Is present only when symptoms are present	2.56 (2.42–2.70)	2.56 (2.37–2.74)	0.97
Can be cured with therapy	2.65 (2.52–2.79)	2.62 (2.43–2.81)	0.74
Requires me to drink fluids especially when thirsty	2.71 (2.58–2.85)	2.72 (2.54–2.89)	0.96
Can occur silently (without signs or symptoms)	2.00 (2.00–2.00)	1.98 (1.94–2.02)	0.21
Is likely to shorten my life (cause premature death)	3.21 (3.12–3.18)	3.17 (3.03–3.30)	0.54
Drugs work best when I have symptoms	2.61 (2.49–2.7))	2.57 (2.37–2.76)	0.70
Can get worse by my lifestyle behaviors or actions	3.09 (2.99–3.20)	3.08 (2.91–3.24)	0.87
Can be disabling	3.24 (3.14–3.33)	3.09 (2.93–3.25)	0.10
Is a threat to my health	3.24 (3.15–3.33)	3.03 (2.87–3.20)	0.02
Needs treatment if I feel fine	3.09 (3.00–3.18)	2.94 (2.78–3.11)	0.09
May improve with drugs and a lot of time	3.08 (3.01–3.15)	2.98 (2.82–3.14)	0.20
Plan of care (drugs, diet...) must be followed forever	2.99 (2.87–3.11)	2.89 (2.69–3.08)	0.35

*SBP* systolic blood pressure, *CI* confidence interval. Responses ranged from 1 = strongly disagree, 2 = disagree, 3 = agree, 4 = strongly agree

**Table 5:** Perceived medication adherence and medication self-efficacy among patients with a history of HTN who presented with normal vs. elevated systolic BP

	Normal SBP mean (95% CI)	High SBP mean (95% CI)	*p* value
1. When you are busy at home	2.47 (2.26–2.69)	2.45 (2.17–2.74)	0.92
2. When you are at work	4.10 (3.69–4.51)	3.69 (3.15–2.24)	0.23
3. When there is no one to remind you	2.50 (2.45–2.67)	2.60 (2.28–2.91)	0.45
4. When you worry about taking them for the rest of your life	2.46 (2.24–2.68)	2.51 (2.22–2.79)	0.79
5. When they cause some side effects	2.37 (2.14–2.60)	2.43 (2.14–2.72)	0.72
6. When they cost a lot of money	2.46 (2.21–2.71)	2.55 (2.23–2.86)	0.68
7. When you come home late from work	3.85 (3.44–4.28)	3.64 (2.11–4.16)	0.52
8. When you do not have any symptoms	2.55 (2.30–2.80)	2.53 (2.24–2.82)	0.90
9. When you are with family members	2.92 (2.21–3.64)	2.62 (2.34–2.90)	0.52
10. When you are in a public place	2.57 (2.35–2.80)	2.62 (2.34–2.90)	0.80
11. When you are afraid of becoming dependent on them	3.00 (2.28–3.71)	2.56 (2.28–2.85)	0.36
12. When you are afraid they may affect your sexual performance	3.39 (3.02–3.76)	3.16 (2.70–3.62)	0.44
13. When the time to take them is between your meals	2.56 (2.32–2.81)	2.53 (2.23–2.82)	0.86
14. When you feel you do not need them	2.58 (2.36–2.81)	2.51 (2.22–2.79)	0.68
15. When you are traveling	2.53 (2.30–2.76)	2.40 (2.10–2.70)	0.50
16. When you take them more than once a day	2.60 (2.37–2.82)	2.55 (2.61–2.83)	0.78
17. If they sometimes make you tired	2.53 (2.30–2.77)	2.45 (2.16–2.74)	0.67
18. When you have other medications to take	2.58 (2.36–2.81)	2.51 (2.22–2.80)	0.68
19. When you feel well	2.58 (2.36–2.81)	2.53 (2.34–2.82)	0.76
20. If they make you want to urinate while away from home	2.63 (2.39–2.87)	2.44 (2.14–2.73)	0.31
21. Get refills for your medications before you run out	2.48 (2.24–2.72)	2.44 (2.14–2.73)	0.81
22. Make taking your medications part of your routine	2.55 (2.32–2.78)	2.58 (2.30–2.87)	0.87
23. Fill your prescriptions whatever they cost	2.49 (2.25–2.73)	2.49 (2.20–2.78))	0.99
24. Always remember to take your BP medications	2.54 (2.31–2.77)	2.55 (2.26–2.83)	0.97
25. Take your BP medications for the rest of your life	2.54 (2.31–2.77)	2.53 (2.24–2.81)	0.95

*SBP* systolic blood pressure, *CI* confidence interval. Responses ranged from: 1 = not at all sure, 2 = somewhat sure, 3 = very sure

## Discussion

In this convenience sample of patients presenting to the UHWI, approximately 2 out 5 patients had an elevated BP in the emergency department, more than the previously reported 1 out of 5 among American patients primarily of African American descent [9]. Moreover, among those with a previous diagnosis of HTN, BP was elevated in more than 60%. Although these data were derived from a cross-sectional sample in an emergency setting, they suggest that a large number of individuals presenting to the ED have elevated systolic blood pressure with an underlying diagnosis of HTN—more than would be expected based on other population-level surveys conducted in the general Jamaican population [2, 8].

Those with a diagnosis of HTN did not differ in their illness beliefs whether they had normal or elevated presenting BP in the ED. These findings conflict with previous studies reporting negative illness beliefs among those with poorly controlled BPs [20, 21]. Pickett et al. [21] evaluated a cohort of 111 outpatient African American patients and noted that patients with poor disease understanding had poorly controlled BPs. Moreover, we found no difference in reported medication self-efficacy among hypertensives with elevated BPs compared to those with normal BPs. A review of the literature reveals a mostly negative correlation, regarding the relationship between disease understanding and medication self-efficacy and the impact on BP control among some populations [22–25].

This disparity between our findings and that previously discussed in the literature may be secondary to this populations’ rote knowledge, but not a true understanding of the disease and importance of BP control. Notably, the majority of these studies evaluating illness beliefs were conducted in an outpatient non-emergent setting. Financial insecurity may also account for this incongruence between disease beliefs and medication self-efficacy [26, 27]. Even so, there was no significant discrepancy between incomes among those presenting with elevated BP compared to those presenting with normal BP. However, our findings that education level is associated with BP control underscores the previously identified association between low literacy rates and elevated ED BP [11, 24, 28]. The ED could play an important role in increasing knowledge surrounding how to achieve well-controlled HTN by providing brief education and referral [13].

While this study provides important, previously understudied, epidemiological information about patients who present to the ED with elevated BP in Jamaica, there are several limitations. We used a cross-sectional design with convenience sampling. We attempted to overcome this by capturing patients presenting to the ED during all times and in all areas of the department; however, based on the sampling methodology, our findings might not be generalizable to the overall ED population. We used previously validated survey tools on illness beliefs and medication adherence self-efficacy, but the validation was not done in an Afro-Caribbean population and these scales may not accurately reflect true perspectives of our target population [17, 18]. Because of interview time constraints, we captured only the illness beliefs of those patients presenting with a previous diagnosis of hypertension. Future studies with larger sample sizes that enroll patients presenting to academic as well as local community hospitals would be of value. Additionally, for simplicity, we did not use a diastolic BP cutoff to define elevated BP; therefore, our estimates of elevated BP frequency may be lower than the true estimate.

## Conclusions

In this single-center study, two of every five Jamaican patients have an elevated BP at ED presentation. Among those with a history of HTN, 60% had an elevated presenting BP. The ED can be an important location to identify patients with chronic disease in need of greater disease-specific education. Those with lower levels of education may be a particularly important population to target. Future studies should evaluate if brief interventions provided by ED medical staff improve HTN control in this patient population.

## Additional file

Additional file 1: HTN JA patient instrument. (DOCX 110 kb)

## Abbreviations

BP: Blood pressure
CI: Confidence interval
DBP: Diastolic blood pressure
ED: Emergency department
HTN: Hypertension
SBP: Systolic blood pressure
SD: Standard deviation
