# Supplementary material for: Elevated blood pressure and illness beliefs: a cross-sectional study of emergency department patients in Jamaica
Source: Int J Emerg Med. 2018 May 30;11:30. doi: 10.1186/s12245-018-0187-6 (PMC5976560; doi:10.1186/s12245-018-0187-6)
Supplement: Supplementary file 1 — HTN JA patient instrument. (DOCX 110 kb) [file 12245_2018_187_MOESM1_ESM.docx]

**Elevated Blood Pressure and Its Consequences in Jamaican Patients: An evaluation of the Emergency Department at the University Hospital of the West Indies**

**Data Collection Instrument** (to be administered by the research investigator)

**Please answer the following questions as completely as possible**

**Patient Demographics**

1. Study Number________________________ Reg #: _________
2. Date ____________________________________
3. Age (years)_______________________________
4. Gender: Male / Female
5. Chief Complaint_______________________________
6. Height___________ Weight __________ BMI_____________ WC­­­­­­­­­­­­­­­­­_________
7. Blood Pressure 1. _________________ 2____________________

**Socio-economic Status**

1. Highest level of Education: [ ] Less than high school [ ] High school/second. School [ ] Bachelors Degree [ ] Masters Degree [ ] Doctorate

[ ] Professional (MD, DDS, Trained teacher)

1. Employed [ ] no [ ] yes [ ] Pension ;NHF [ ] yes [ ] no
2. What is your weekly household Income in Jamaican Dollars (total sum of money earned by all adults in home? Write the actual figure.

**Hypertension Status**

1. Have you ever been told that you have high blood pressure? [ ] Yes [ ] No

If no and blood pressure normal you will not be asked any more questions and thanks for participation in the study.

If no and pressure is high proceed to causes of high blood pressure the questions numbered 19 on page 15.

If yes please continue answering the questions in sequence.

1. How long have you known that you have high blood pressure____________years?
2. Have you been prescribed medication for your hypertension [ ] Yes [ ] No
3. Do you take your medication as prescribed for your hypertension? [ ] Yes [ ] No
4. What medications do you take for your high blood pressure_____________________________________________________________________________________________________________________

**Utilization of the ED for Blood Pressure Control**

1. Where do you usual go to have your high blood pressure checked and have new prescriptions written?

[ ] Health Center [ ] Private Doctor [ ] Hospital clinic

[ ] Accident and Emergency department/casualty

1. When was the last time you saw a health professional for your high blood pressure?

| [ ] One week ago | [ ] One month ago |
| --- | --- |
| [ ] Two months ago | [ ] Three months ago |
| [ ] Less than six months ago | [ ] One to five years ago |
| [ ] Six months to one year ago | [ ] More than five years ago |

**Survey of Illness Beliefs in Hypertension**

Directions: This part of the survey provides 14 statements that might describe what hypertension means to you, including your beliefs and understanding about hypertension. The purpose is to learn what you believe about high blood pressure. Read each statement then circle the best response:

| a. Is an illness that I cannot influence by my behaviour. | **SD** | **D** | **A** | **SA** | **DK** |
| --- | --- | --- | --- | --- | --- |
| b. Is something I go “in” and “out” of. | **SD** | **D** | **A** | **SA** | **DK** |
| c. Is present only when symptoms are present. | **SD** | **D** | **A** | **SA** | **DK** |
| d. Can be cured with drugs and other therapies. | **SD** | **D** | **A** | **SA** | **DK** |
| e. Requires me to drink fluids, especially when I feel thirsty | **SD** | **D** | **A** | **SA** | **DK** |
| **f.** Can occur silently (without signs or symptoms). | **SD** | **D** | **A** | **SA** | **DK** |
| **g.** Is likely to shorten my life (cause premature death). | **SD** | **D** | **A** | **SA** | **DK** |
| **h.** Drugs work best when I have symptoms. | **SD** | **D** | **A** | **SA** | **DK** |
| i. Can get worse by my lifestyle behaviors or actions | **SD** | **D** | **A** | **SA** | **DK** |
| j. Can be disabling. | **SD** | **D** | **A** | **SA** | **DK** |
| k. Is a threat to my health? | **SD** | **D** | **A** | **SA** | **DK** |
| l. Needs treatment even if I feel fine | **SD** | **D** | **A** | **SA** | **DK** |
| m. May improve with drugs and a lot of time. | **SD** | **D** | **A** | **SA** | **DK** |
| n. Plan of care (drugs, diet…) must be followed forever | **SD** | **D** | **A** | **SA** | **DK** |

**Strongly disagree (SD) disagree (D) agree (A) or strongly agree (SA)**, **or do not know (DK)**

Choose *strongly* disagree or *strongly* agree if you are very certain of your view about the statement is something you may or may not believe.

Answer each statement as best as you can. It is important for you to respond to each statement based on your actual beliefs and not on how you think you should respond to each statement.

**Medication Self-Efficacy Assessment**

For each of the situations listed below, please rate how sure you are that you can take your blood pressure medications **Most of the time.**

|  | Not At All Sure | Somewhat Sure | Very Sure |
| --- | --- | --- | --- |
| 1. When you are busy at home |  |  |  |
| 1. When you are at work |  |  |  |
| 1. When there is no one to remind you |  |  |  |
| 1. When you worry about taking them for the rest of your life |  |  |  |
| 1. When they cause some side effects |  |  |  |
| 1. When they cost a lot of money |  |  |  |
| 1. When you come home late from work |  |  |  |
| 1. When you do not have any symptoms |  |  |  |
| 1. When you are with family members |  |  |  |
| 1. When you are in a public place |  |  |  |
| 1. When you are afraid of becoming dependent on them |  |  |  |
| 1. When you are afraid they may affect your sexual performance |  |  |  |
| 1. When the time to take them is between your meals |  |  |  |
| 1. When you feel you do not need them |  |  |  |
| 1. When you are travelling |  |  |  |
| 1. When you take them more than once a day |  |  |  |
| 1. If they sometimes make you tired |  |  |  |
| 1. When you have other medications to take |  |  |  |
| 1. When you feel well |  |  |  |
| 1. If they make you want to urinate while away from home |  |  |  |
| 1. Get refills for your medications before you run out |  |  |  |
| 1. Make taking your medications part of your routine |  |  |  |
| 1. Fill your prescriptions whatever they cost |  |  |  |
| 1. Always remember to take your blood pressure medications |  |  |  |
| 1. Take your blood pressure medications for the rest of your life |  |  |  |

1. **Causes of Hypertension**

Which of the following do you think **causes** high blood pressure? (Multiple responses allowed), DK = Don’t know

Diet [ ] Yes [ ] No [ ] DK High cholesterol [ ] Yes [ ] No [ ] DK

Exercise [ ] Yes [ ] No [ ] DK Cigarette smoking [ ] Yes [ ] No [ ] DK

Sugar Intake [ ] Yes [ ] No [ ] DK Family History [ ] Yes [ ] No [ ] DK

Salt intake [ ] Yes [ ] No [ ] DK Alcohol [ ] Yes [ ] No [ ] DK

Caffeine [ ] Yes [ ] No [ ] DK Lack of Sleep [ ] Yes [ ] No [ ] DK

Stress [ ] Yes [ ] No [ ] DK Obesity [ ] Yes [ ] No [ ] DK

1. What are possible **consequences** of high blood pressure? DK = Don’t know

| **Consequence** |  |
| --- | --- |
| Cholesterol buildup | [ ] Yes [ ] No [ ] DK |
| Cancer | [ ] Yes [ ] No [ ] DK |
| Heart Attack | [ ] Yes [ ] No [ ] DK |
| Diabetes | [ ] Yes [ ] No [ ] DK |
| Kidney Failure | [ ] Yes [ ] No [ ] DK |
| Heart Failure | [ ] Yes [ ] No [ ] DK |
| Stroke | [ ] Yes [ ] No [ ] DK |
| Ulcers | [ ] Yes [ ] No [ ] DK |
| Muscle Weakness | [ ] Yes [ ] No [ ] DK |
| Heart Muscle Thickening | [ ] Yes [ ] No [ ] DK |
| Confusion & Headaches | [ ] Yes [ ] No [ ] DK |

**Evidence of Target Organ Injury**

**21. *Presenting Complaint*** _________________________________

**22. History of Target Organ Injury** **including Chart Review**

Presence of any of the following target-organ diseases at baseline as noted by self- reported history or as documented in the medical records:

Heart failure [ ] yes [ ] no

Left ventricular hypertrophy [ ] yes [ ] no

Aortic dissection [ ] yes [ ] no

Acute myocardial infarction [ ] yes [ ] no

Stroke or transient ischemic attack (TIA) [ ] yes [ ] no

Chronic kidney disease [ ] yes [ ] no

Renal failure [ ] yes [ ] no

**23 Evidence of new target-organ injury**

- 1. Stroke/TIA [ ] yes [ ] no

Limb weakness [ ] Slurred speech [ ] Loss of vision [ ] other

CT scan done [ ] yes [ ] no

Carotid Ultrasound [ ] yes [ ] no

- 1. Heart failure [ ] yes [ ] no
  2. Kidney failure [ ] yes [ ] no

Electrolytes [ ] yes [ ] no

Creatinine [ ] yes [ ] no

d. Acute MI

Cardiac Enzymes [ ] yes [ ] no

Troponin [ ] yes [ ] no

B -naturetic peptide [ ] yes [ ] no

ECG [ ] yes [ ] no

Chest X-ray [ ] yes [ ] no

Echo [ ] yes [ ] no

EF<50% [ ] yes [ ] no

Thrombolytics [ ] yes [ ] no

Intravascular procedures

Stenting [ ] yes [ ] no

Embolectomy [ ] yes [ ] no

Operative intervention [ ] yes [ ] no

Aortic Dissection [ ] yes [ ] no

Chest CT Scan [ ] yes [ ] no

1. Peripheral Vasc dz

Peripheral pulses [ ] yes [ ] no

Sensation [ ] yes [ ] no

1. Vision

Fundoscopy [ ] yes [ ] no

Working diagnosis __________________________________________________

**24. Disposition**: - Admitted to hospital [ ] yes [ ] no

Admitted to ICU [ ] yes [ ] no

Admitted to General Hospital Ward [ ] yes [ ] no

Discharged to home from the ED [ ] yes [ ] no

Died [ ] yes [ ] no
